# Modelling the health and economic impact of sugary sweetened beverage tax in Canada

**DOI:** 10.1371/journal.pone.0277306

**Published:** 2022-11-10

**Authors:** Siyuan Liu, Paul J. Veugelers, Katerina Maximova, Arto Ohinmaa

**Affiliations:** 1 Population Development Studies Center, Institute of Health Science, School of Sociology and Population Studies, Renmin University of China, Beijing, China; 2 Population Health Intervention Research Unit, School of Public Health, University of Alberta, Edmonton, Alberta, Canada; 3 MAP Centre for Urban Health Solutions, St. Michael’s Hospital, Toronto, Ontario, Canada; 4 Dalla Lana School of Public Health, University of Toronto, Toronto, Ontario, Canada; PAHO/WHO, UNITED STATES

## Abstract

**Background:**

With the increasing concerns about the health and economic burden attributed to sugar-sweetened beverages (SSBs) consumption, SSB taxation has been proposed and implemented in many countries. Many previous economic evaluations of SSB taxation have shown that this kind of policy is cost-effective. However, the magnitude of impact varies. This study aims to design a comprehensive model to estimate the impact and cost-effectiveness of the SSB tax in Canada.

**Methods:**

A proportional multi-state life table-based Markov model was chosen to estimate the impacts of SSB tax in Canada. The health-related quality of life (including disability-adjusted life years (DALYs) and quality-adjusted life years (QALYs)), the costs (including health care costs and intervention costs), and the tax revenue were the main health and economic outcomes. We compared the simulated SSB tax with the current practice from the public health care payer perspective, and the tax was applied to the 2015 adult Canadian population up to 100 years. The economic model was built following guidelines from the Canadian Agency for Drugs and Technologies in Health.

**Results:**

After implementing a CAD$0.015/oz SSB tax, 282,104 cases of overweight and obesity, 210,542 cases of diseases, and 2,189 deaths could be prevented. The simulated SSB tax has the potential to avert 2.3 million DALYs, gain 1.5 million QALYs, and save CAD$32,583 million in health care costs in a lifetime period. The incremental cost-effectiveness ratio for the SSB tax was CAD$ -24,933/QALY. The SSB tax with different tax levels (CAD$0.01/oz and CAD$0.02/oz) remained cost-effective.

**Conclusion:**

Implementing the SSB tax in Canada is a potential cost-effective policy option for reducing obesity and related chronic diseases. The model built in this study provides a more accurate estimate of health and economic impact of SSB tax and could be used to estimate other sugar tax options.

## Introduction

Unhealthy eating is a leading risk factor for obesity and related chronic diseases (CDs), such as diabetes mellitus type 2 (DM2) [1]. It contributes to 32% of deaths and 21% of the global risk-attributable disease burden and results in CAD$15.8 billion annual costs (including direct health care costs and indirect costs) in the treatment and management of CDs in Canada [2–4]. In order to decrease the health and economic burden, cutting down on unhealthy foods (e.g., saturated fat, processed meats, and sugar-sweetened beverages (SSBs)) and promoting the consumption of healthy foods (e.g., vegetables, fruit, and nuts) have become the top priority.

The SSBs are beverages containing free sugars, including carbonated soft drinks, ready-to-drink sweetened tea and coffee, energy drinks, sports drinks, flavoured bottled water, sweetened milk and drinkable yogurt, fruit drinks, and 100% fruit juice [5]. Among dietary risk factors, the attributable burden of high SSBs consumption significantly increased over the past decades [2]. This increase has attracted substantial public attention to take action to reduce SSBs consumption. Many countries have implemented SSBs reduction policy programs, such as SSB tax, public awareness initiatives, and SSB labelling policies [6–8]. Research suggests that these interventions could increase SSBs prices and reduce SSBs consumption [9].

To date, there are over 40 countries that have implemented taxation of SSBs [10–12]. For example, Mexico implemented a 1 peso per liter excise tax on SSBs [6]. Compared with other sugar-reducing policy options, the taxation strategy has been shown to be a cost-effective and feasible priority intervention [13–15]. Empirical and simulation studies have shown that the SSB tax could effectively reduce obesity and related CDs, improve the health-related quality of life (HRQoL), save health care costs and generate tax revenues [10, 16–18].

Although all previous simulation studies have a consistent conclusion that SSB tax is cost-saving, the magnitude of health and economic impacts of SSB taxes varied [18]. Many factors (e.g., design of SSB tax, target population, time horizon, modelling methods, and parameters) could lead to differences among studies. For example, SSB tax simulation studies with higher SSB tax levels, longer periods, and larger study populations tend to show larger health and economic outcomes [18]. Price elasticities and CDs included in the model were two important parameters. Using a set of reliable price elasticities and considering as many CDs as possible could make the simulated changes in consumer behaviours and health outcomes close to the real-world setting.

However, most previous SSB tax simulation studies used a relatively short period, considered a limited number of substitutes (e.g., water, plain milk, and diet beverages) and included a few CDs related to SSBs [19, 20]. Two Canadian studies have built simulation models that included 23 CDs to evaluate the impact of a 20% SSB tax in a lifetime period [19, 21]. They also missed six CDs in the Global burden of disease (GBD) report and did not consider substitutes for other sugary foods. To improve the above-mentioned limitations of the model, in this study, we objective to 1) build and introduce a new detailed SSB tax simulation model and 2) estimate the health and economic impact and cost-effectiveness of SSB taxes in Canada using this model.

## Methods

### Model overview

We built a proportional multi-state life table-based Markov model to simulate the impacts of SSB tax on the 2015 Canadian adult population over their lifetime from the public health care payer perspective. The “business as usual” was modelled as a comparator based on the guidelines from the Canadian Agency for Drugs and Technologies in Health (CADTH) [22]. Following the CADTH, we set the discount rate of health outcomes (e.g., disability-adjusted life years (DALYs) and quality-adjusted life years (QALYs)) and cost outcomes (including health care costs, tax revenue and intervention costs) as 1.5%. Model quality was checked using the Consolidated Health Economic Evaluation Reporting Standards (CHEERS) [23]. The CHEERS Checklist is shown in the S1 Table in S1 File.

The baseline population was modelled as a closed cohort that replicated the 2015 adult Canadian population (20 years and above). The population aged over time until everyone was dead or reached the age of 100 years. Three parameters: population size, all-cause mortality rate, and the rate of prevalence years lived with disability (pYLD) were used to build the life table. These parameters were interpolated to 1-year age and sex group using the epidemiology software DisMod II (EpiGear, Version 1.05, Brisbane, Australia) based on the data from Statistic Canada [24, 25] and the Global Burden of Disease (GBD) Results Tool [26]. The life table only considers the age and sex of the population, ignoring other characteristics, such as education, ethnicity, and income.

**Fig 1:**
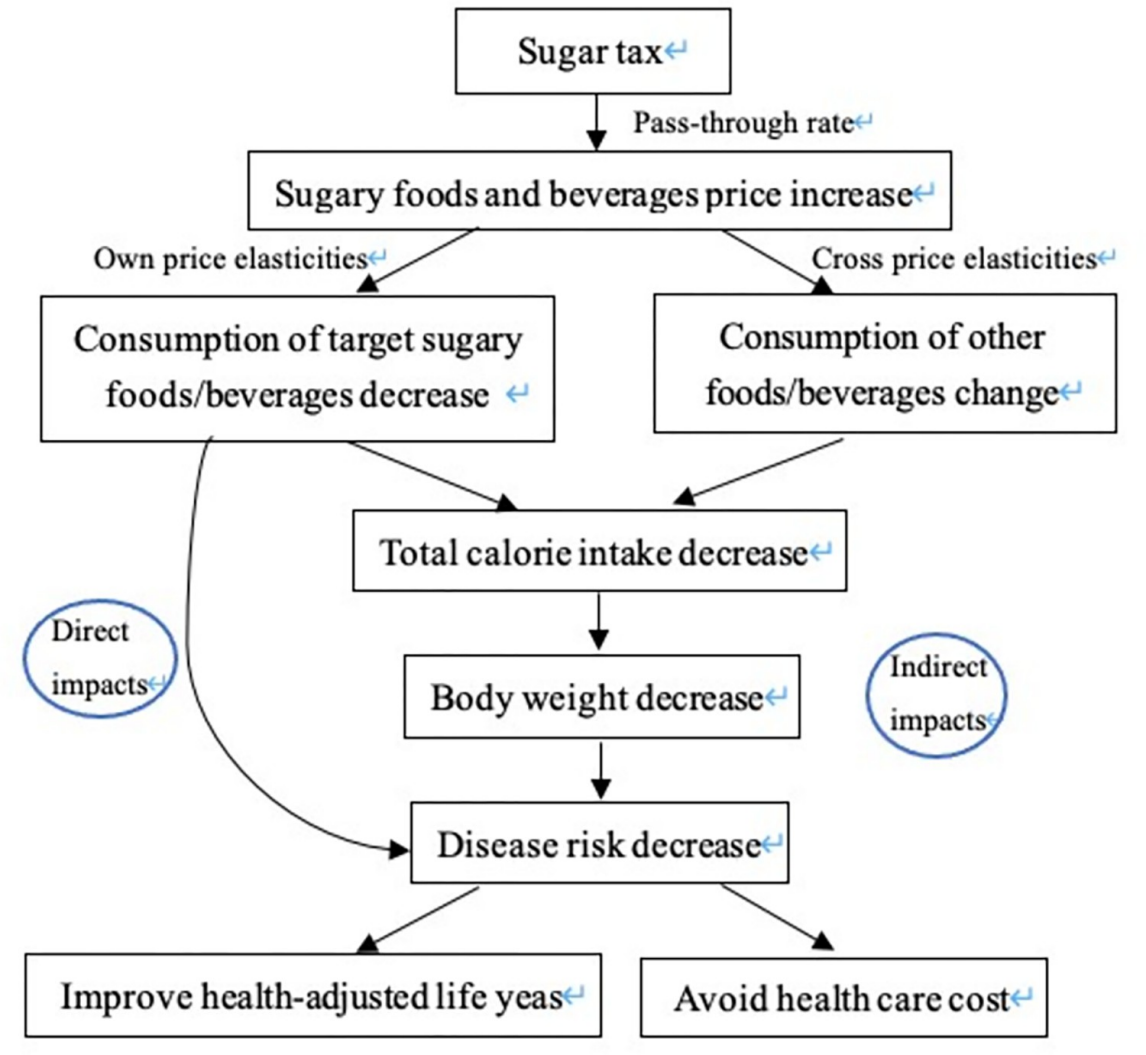
The model diagram of a simulated sugar tax for the intervention population.

The model diagram of sugar taxes for the intervention population is depicted in Fig 1. Taxation design, model selection, detailed parameters (e.g., pass-through rate and price elasticities), data sources and outcomes of the model are described in the next sections. The University of Alberta Research Ethics Board approved this study (Pro00073295).

### Intervention specification

In this study, we simulated three flat taxes levied on the weight of SSBs: CAD$0.01/oz, CAD$0.015/oz, and CAD$0.02/oz tax on all sugary beverages. We chose the tax based on weight of SSBs because it was the most commonly used type of SSB tax in the real-world setting and was relatively easy to manage and adjust according to inflation [27]. The tax levels were set based on the implemented SSB taxes in the UK (CAD$0.01/oz), Portugal (CAD$0.01/oz), and the US (ranging from CAD$0.01/oz to CAD$0.02/oz) [13, 27]. The original tax levels of these SSB taxes from other countries were converted to Canadian dollar values using the web-based tool CCEMG-EPPI-Centre Cost Converter [28]. The CAD$0.015/oz tax on SSBs was set as the base case, while all other taxes were shown in the scenario analysis.

### Model type selection

**Fig 2:**
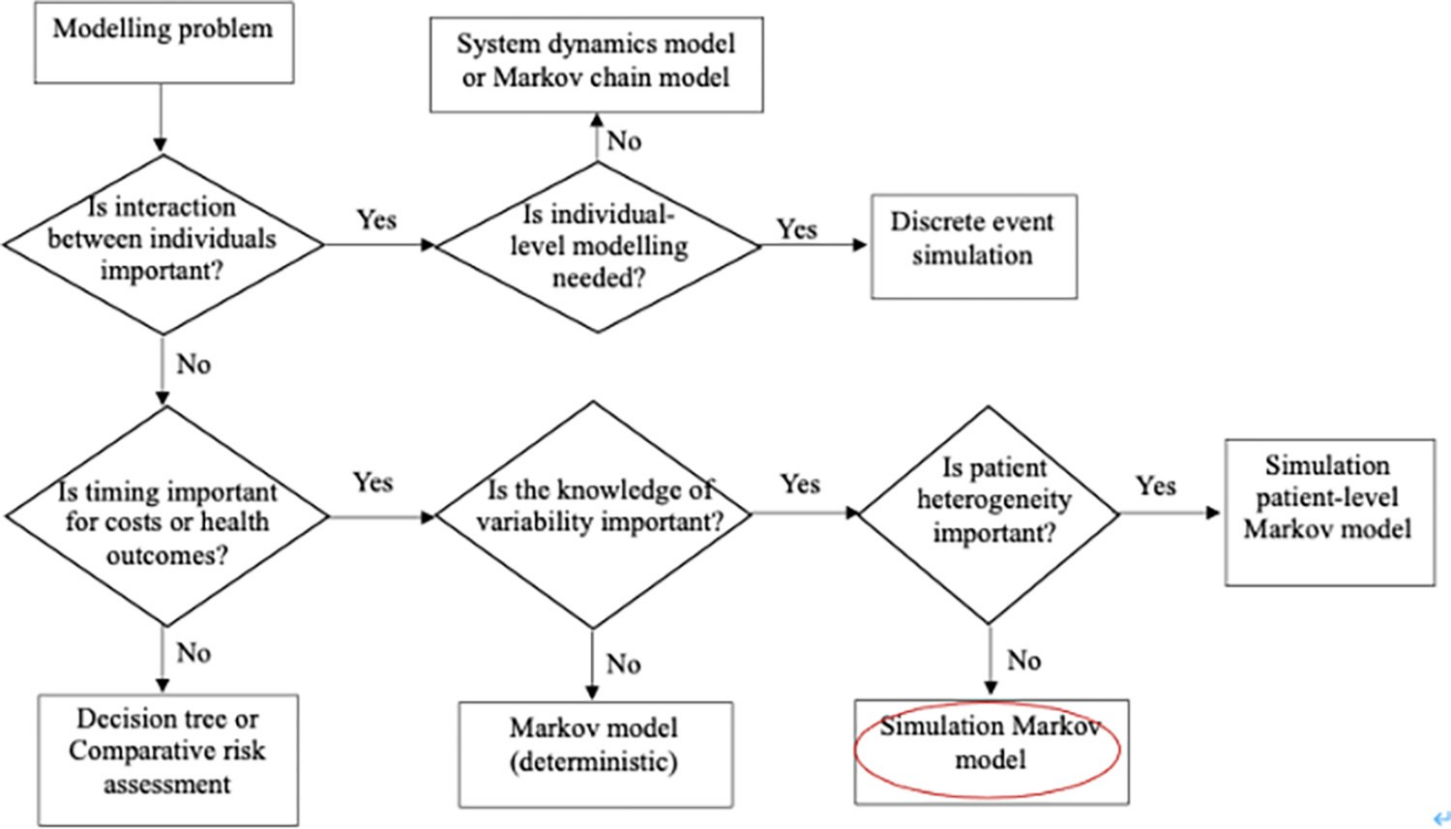
Model type selection flowchart for the economic evaluation of a sugar tax. Adapted from Brennan et al. [30].

There are various available model structures for the economic evaluation of sugar taxes [29]. In this study, we determined the model type using a flowchart adapted from Brennan et al. (Fig 2) [30]. Selection questions in Brennan et al.’s study were revised based on the taxonomy of model structure put forward by Barton et al. [31]. Questions related to interactions between individuals, the timing for costs or health outcomes, the knowledge of variability, and patient heterogeneity were considered in the selection process. Because the simulated SSB tax has long-term impacts on disease risks, preference of decision-makers to know the variability in the results, and the differences in the history of diseases and co-morbidity are not important for this study, the simulation Markov model was selected as the most suitable model type for this study (Fig 2).

In addition, to increase the number of possible disease states, we combined the multistate life tables with Markov models. The transition probabilities were assumed to not change with the past states or time. Although the cohort state-transition model is restricted by the Markovian property, the multistate life table is a reasonable choice to simulate the effects of SSB tax [32, 33]. Furthermore, state-transition models are simple to develop, debug, communicate, analyze, and readily accommodate the evaluation of parameter uncertainty [32, 34].

### Change in price of SSBs

After implementing the SSB tax, the manufacturers, distributors, and retailers would pass the sugar tax to consumers by increasing the product prices [35, 36]. The pass-through rate was expected to be heterogeneous across brands, retail groups, package sizes, and regions because companies and retailers might absorb some of the tax cost or increase the product price above the tax rate [35, 36]. Based on the price change of SSBs in Mexico [35] and France [36] and the pass-through rate used in the previous SSB tax simulation [18], we assumed the SSB tax would be 100% pass-through to consumers. The 80% and 120% pass-through rates were modelled in the sensitivity analysis. According to SSBs’ prices on a Canadian grocery store website [37] and the consumer price index [38], we estimated the model baseline average SSBs prices in Canada to be CAD$0.10/oz. The CAD$0.01/oz, CAD$0.015/oz, and CAD$0.02/oz tax would increase around 10%, 15%, and 20% of SSBs prices, respectively.

### Change in consumption of SSBs and energy intake

The baseline consumption of SSBs and energy intake of Canadians were estimated based on the 24-hour dietary recalls of the 2015 Canadian Community Health Survey (CCHS)–Nutrition [39]. We estimated the mean and standard error of SSBs consumption and energy intake per day per person for each 10-year age and sex sub-group. Proportional sampling weights were applied to ensure estimates were representative of all residents of Canada.

Due to a lack of evidence about the long-term change in consumption of SSB and total energy intake (TEI) after implementing the SSB tax, we assumed that they changed as a one-time reduction in one year and lasted for a lifetime. It was in line with the previous SSB tax model [18]. We used both the own- and cross-price elasticities of demand to measure the changes in consumption corresponding to the changes in the price of SSBs. The own- and cross-price elasticity represents the proportional change in the purchase of a product in response to a 1% increase in its price and the price of other products, respectively.

Because Canadian price elasticities were not available for most food categories consumed by Canadians [40], we used more recent own- and cross-price elasticities from New Zealand (S2 Table in S1 File) [41]. The price elasticities from New Zealand have comprehensive food categories, which include nearly all food categories consumed by Canadians [42]. Another reason for applying the price elasticates from New Zealand to the current study was that both New Zealand and Canada are classic immigrant countries with similar histories (i.e., British colonization) and economic levels. Although foreign price elasticities have been adapted in the previous SSB tax evaluations [17, 21], we also applied price elasticities from the US [43] in sensitivity analysis to estimate the changes in outcomes due to the choice of different price elasticities. The price elasticities from the US are also robust, but they lack some food groups (e.g., fresh meat, fresh vegetables, pasta, grains and flour, oil, and pastry cook products).

### Change in body mass index

We extracted the baseline consumption and body mass index (BMI) by age and sex from the 2015 CCHS–Nutrition data and weighted them by the 2015 Canadian population. Proportional weights were calculated using the weight variable for measured height and weight. Considering the temporal change in BMI, we incorporated the model with the predicted BMI trends (S3 Table in S1 File) [44]. The changes in BMI attributed to the SSB tax were calculated using the energy equation in Swinburn et al.’s studies [45, 46]. The prevalence and prevented cases of overweight (25<BMI≤30) and obesity (BMI>30) due to SSB taxes were also estimated.

### Change in disease risks

In the simulation model, we included 27 BMI-related and two non-BMI-related CDs [2]. The CDs mediated by the BMI include DM2, cancers (e.g., breast cancer and thyroid cancer), cardiovascular diseases (e.g., ischaemic heart disease and hemorrhagic stroke), and other conditions (e.g., gallbladder and biliary diseases). The non-BMI-related CDs are ischaemic heart disease and DM2. The complete lists of these CDs are presented in the S4 Table in S1 File.

Relative risks (RRs) of these diseases for people aged 20 and above were from the 2017 GBD [2]. The potential impact fraction (PIF), defined as the proportional changes in disease risk due to change in exposure to a related risk factor, was used to estimate the changes in disease incidence attributed to the changes in BMI or SSBs consumption [47]. Microsoft Excel and add-ins: Risk Factor (EpiGearXL 5.0) from EpiGear (Brisbane, Australia) were used to calculate the PIFs. The detailed formula of PIF was presented as follows.


$$PIF=\frac{\int_{l}^{h}\;RR\;(x)P(x)dx-\int_{l}^{h}\;RR\;(x)P^{*}(x)dx}{\int_{l}^{h}\;RR\;(x)P(x)dx}$$

Where x denotes the exposure levels the risk factor can take on, RR(x) is the RR function, P(x) is the original risk-factor distribution, P*(x) is the risk-factor distribution after the SSB tax, dx denotes that the integration is done with respect to x, and l and h are the integration boundaries [47].

**Fig 3:**
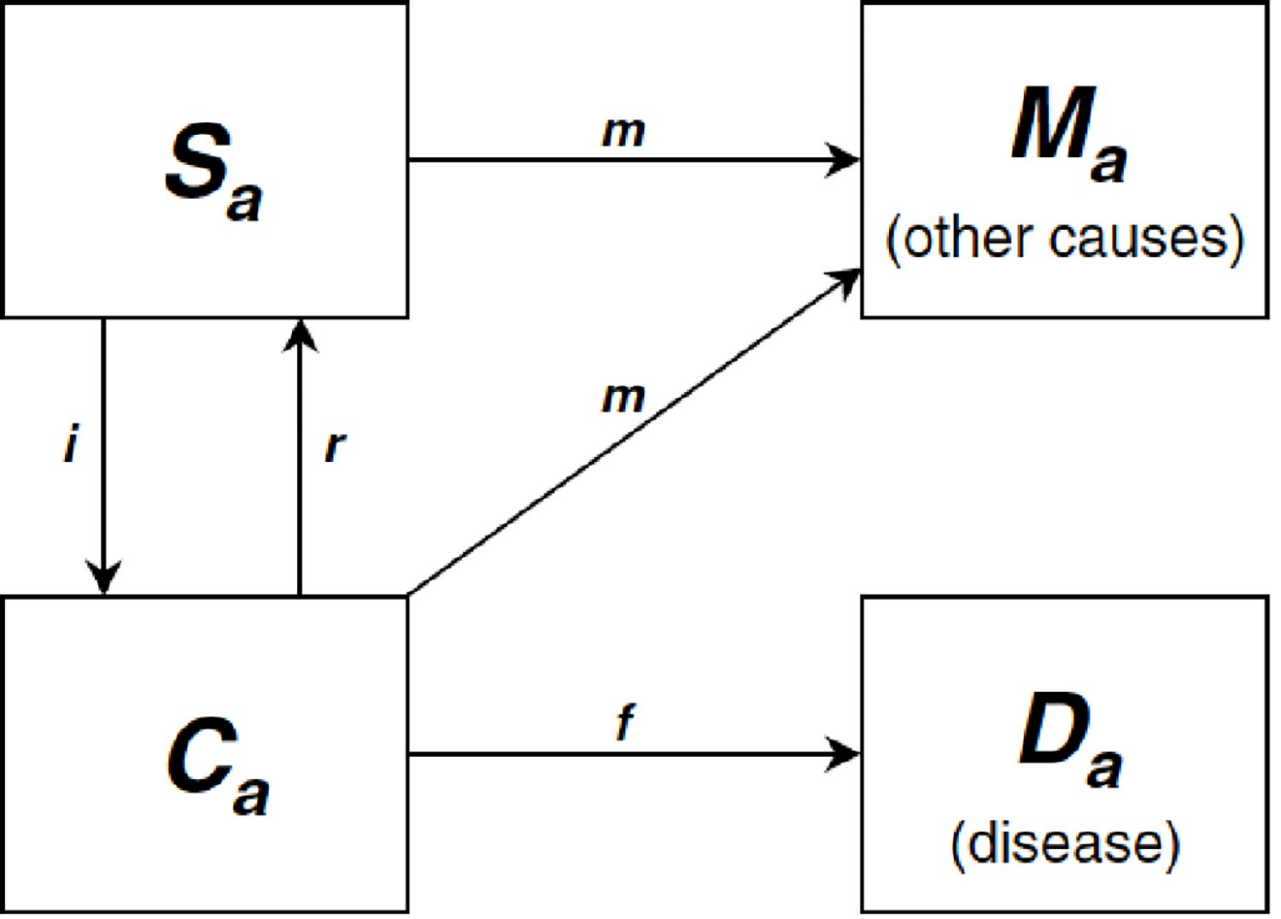
The conceptual disease model. Adapted from Barendregt et al. [54].

Each disease was modelled separately in the model using an established disease model with four states (healthy, diseased, dead from disease and dead from all other causes) [48]. We collected the transition hazards between these four states of incidence, remission, case fatality and mortality from all other causes from the Statistic Canada and the GBD Result Tool (S5 Table in S1 File) [29, 49–53]. These parameters were interpolated and smoothed to 1-year age and sex group using the DisMod II. The conceptual disease model is shown in Fig 3 [54]. Disability weights for DALYs calculation were borrowed from the WHO [55]. Corresponding changes in disease-specific incidence, prevalence and mortality for a lifetime were calculated using the life table model.

### The cost-effectiveness analysis

**Fig 4:**
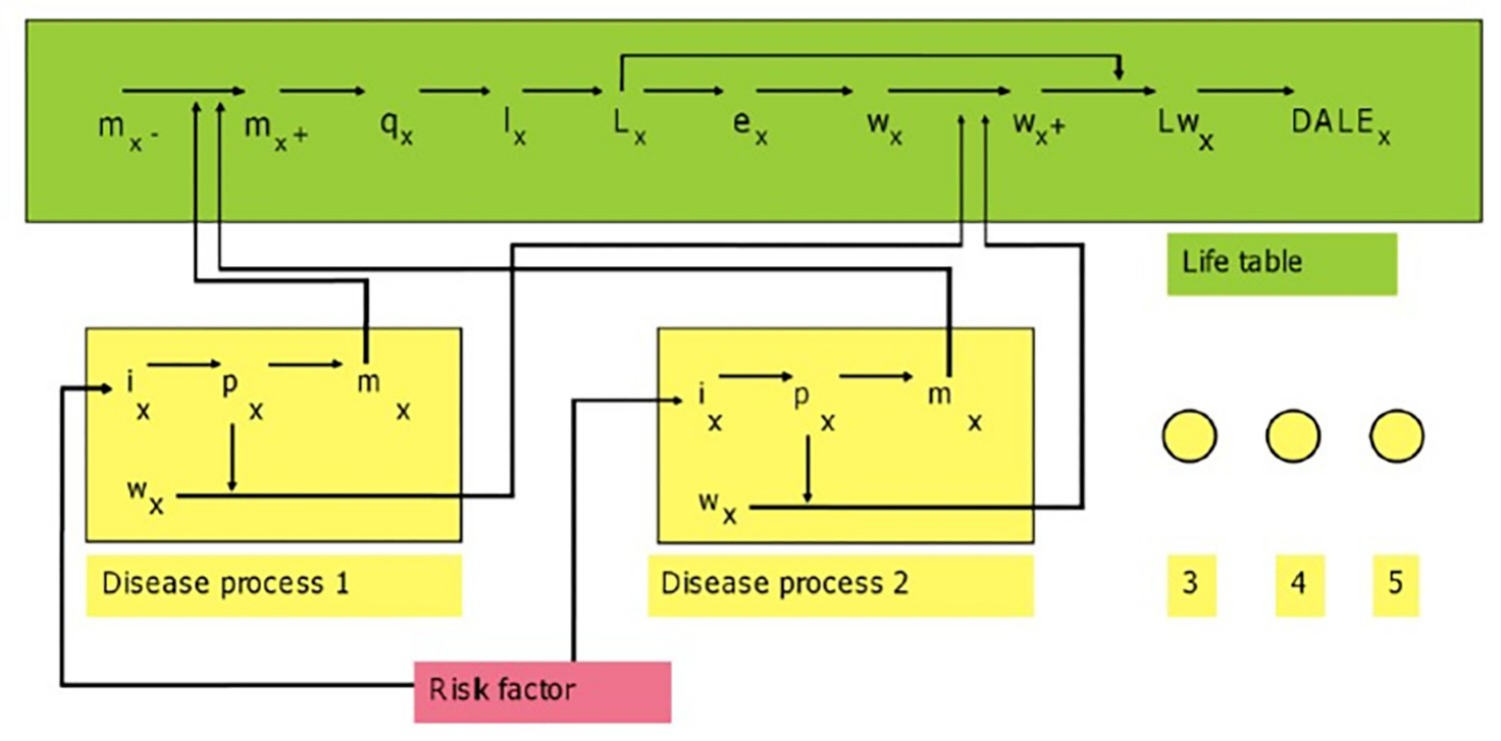
The schematic of a proportional multi-state life table. From Lee et al. [56]. x is age; i is incidence; p is prevalence; m is mortality; w is pYLD; q is probability of dying; l is number of survivors; L is life years; Lw is disability-adjusted life years; e is life expectancy and DALE is disability-adjusted life expectancy, and where ‘-’ denotes a parameter that specifically excludes modelled diseases, and ‘+’ denotes a parameter for all diseases (i.e., including modelled diseases).

Averted DALYs and saved QALYs were estimated in the proportional multi-state life table. Fig 4 shows the schematic of a proportional multi-state life table [56]. We used the disease-specific mortality combined with all other causes of mortality from the population to estimate the overall morbidity and mortality in the total population. The DALYs were constructed from the burden of disease from premature death (years of life lost) and the disabling results of illness (years lived with disability). The QALYs were estimated based on shifts in obesity prevalence and published QALY weights related to obesity by age and sex for adults [57].

The cost outcomes of the simulation model include health care costs, tax revenue and intervention costs. We calculated the differences in the direct health care costs (including hospital care, physician care, drug, other institutions, other professionals, capital, public health, administration, and other health spending) between the interventions and the current practice. Data from the 2010 Economic Burden of Illness in Canada (EBIC) dataset [58] and methods from Jones [19] were used in the calculation. We did not include indirect costs in this study following the previous SSBs tax simulation studies [18]. Detailed health care cost inputs can be found in the S6 Table in S1 File.

Tax revenue was calculated based on the consumption of SSBs. Due to the absence of data regarding the costs of implementing sugar tax in Canada, the intervention costs were 2% of tax revenue based on the previous studies and data from the US operating a sugar tax [17, 59]. All cost estimations were presented in 2015 Canadian dollars [38]. Additionally, we computed the incremental cost-effectiveness ratios (ICERs) for each intervention compared with the no-intervention scenario. The ICERs were calculated by dividing the difference in expected health care costs by the difference in expected health outcomes. We did not include the tax revenue and intervention costs because we used the public health care payer perspective.

### Uncertainty and sensitivity analysis

We conducted a Monte Carlo simulation with bootstrapping (2000 iterations) while incorporating probabilistic uncertainty from model inputs (including mean BMI, RRs, the effect of change in energy intake on weight, free sugar intake and price elasticity of demand) using the Ersatz (Version1.34). Uncertainty intervals (95% uncertainty intervals (UI)) were calculated to reflect parameter uncertainties. In addition, we conducted sensitivity analyses to assess the effect of modifying key assumptions and parameters on the results. First, we assumed the BMI remained at 2015 levels to remove the impact of the BMI trend. Second, we set the pass-through rate as 80% and 120% based on the previous SSB tax simulation models and empirical studies [18, 35, 36]. Third, we set the discount rate at 3% following the previous studies [18]. Lastly, we used price elasticities from the US to test the impact of different price elasticities [43].

## Results

### Health benefits of the SSB tax

After one year of the implementation of a CAD$0.015/oz (base case) SSB tax, the price of SSBs would increase by 15%, and SSBs consumption was estimated to reduce by 17%. Table 1 shows the changes in energy intake from SSBs and TEI. On average, the TEI would decrease by 19.25 kcal per day (95%UI: 18.71, 19.77) for males and 12.27 kcal per day (95%UI: 11.91, 12.61) for females. The SSB tax produced a reduction in BMI of 0.18 (95%UI: 0.18, 0.19) for males and 0.14 (95%UI: 0.14, 0.15) for females. Compared with adults, children and adolescents showed more reduction in SSBs consumption, TEI, and BMI due to SSB tax (Table 1, S7 Table in S1 File).

**Table 1:** Mean change in energy intake from sugar sweetened beverages (SSBs) and total energy intake (TEI) due to a CAD$0.015/oz SSB tax, by age and gender.

Age group^2^	Changes in energy intake from SSBs (per person, per day; kcal)				Changes in TEI (per person, per day; kcal)			
	Females		Males		Females		Males	
	Mean	95%UI^1^	Mean	95%UI	Mean	95%UI	Mean	95%UI
**1~9**	-16.11	(-16.87, -15.33)	-18.39	(-19.27, -17.50)	-14.11	(-14.86, -13.33)	-16.08	(-16.95, -15.21)
**10~19**	-20.73	(-11.71, -19.71)	-33.01	(-34.31, -31.64)	-19.56	(-20.53, -18.56)	-30.50	(-31.92, -29.21)
**20~29**	-16.19	(-17.62, -14.73)	-28.68	(-30.99, -26.39)	-15.23	(-16.72, -13.73)	-28.24	(-30.83, -25.52)
**30~39**	-13.36	(-14.36, -12.33)	-23.76	(-25.64, -21.93)	-12.79	(-13.84, -11.71)	-22.51	(-24.47, -20.56)
**40~49**	-9.98	(-10.91, -9.09)	-17.48	(-19.05, -15.87)	-9.34	(-10.33, -8.37)	-17.45	(-19.09, -15.89)
**50~59**	-9.06	(-9.97, -8.12)	-14.71	(-15.80, -13.60)	-10.35	(-11.39, -9.35)	-14.31	(-15.56, -13.13)
**60~69**	-13.53	(-14.89, -12.17)	-9.27	(-10.11, -8.40)	-8.90	(-9.82, -7.95)	-13.12	(-14.54, -11.68)
**70~79**	-9.33	(-10.19, -8.84)	-11.46	(-12.44, -10.50)	-8.92	(-9.83, -8.05)	-10.35	(-11.37, -9.34)
**80~89**	-11.78	(-13.09, -10.46)	-14.75	(-17.07, -12.40)	-11.46	(-12.84, -10.13)	-14.03	(-16.37, -11.64)
**90+**	-13.16	(-16.07, -10.27)	-17.78	(-24.09, -11.88)	-10.65	(-13.71, -7.62)	-16.07	(-22.84, -9.46)
**Total**	-12.83	(-13.17, -12.52)	-20.27	(-20.77, -19.77)	-12.27	(-12.61, -11.91)	-19.25	(-19.77, -18.71)

^1^ 95%UI, 95% uncertainty interval

^2^ 1~19 age groups were not included in cost-effectiveness modeling.

The SSB tax was estimated to reduce the mean prevalence of overweight and obesity by 0.91% for males and 0.68% for females. After implementing the SSB tax, 160,567 and 121,537 cases of overweight and obesity could be prevented for males and females, respectively, in one year. The simulated SSB tax was projected to prevent a total of 210,542 (95% UI: (195,995, 223,869) new cases of CDs and 2,189 (95% UI: 1,866, 2,447) deaths over the next 25 years (2016–2041). The prevented incident cases and deaths over 25 years for each CD can be found in the S8 Table in S1 File.

### Cost-effectiveness of the SSB tax

Table 2 shows the health impact, costs, and cost-effectiveness of a CAD$0.015/oz SSB tax. As a result of the SSB tax, the adult population was estimated to avert 2.3 million (95% UI: 2.0, 2.6 million) DALYs and save 1.5 million (95% UI: 1.3, 1.7 million) QALYs over a lifetime period. The overall lifetime direct health care cost savings attributable to the SSB tax were CAD$37,548 million (95% UI: CAD$34,155, 39,784 million). Males were estimated to have more health benefits and health care cost offsets than females (Table 1). The SSB taxes was cost-effective with negative ICER (CAD$-24,933/QALY, 95% UI: -27,569, -23,043).

**Table 2:** Health impact, costs, and cost-effectiveness of a CAD$0.015/oz SSB tax in Canada over a lifetime period.

	Females	Males	Females and Males
**Health outcomes**			
DALYs^1^ averted	895,316 (795,638, 992,823)	1,396,057 (1,209,906, 1,566,893)	2,291,373 (2,005,544, 2,559,716)
QALYs^2^ gained	573,750 (520,936, 623,612)	935,599 (824,442, 1,032,175)	1,509,349 (1,345,378, 1,655,787)
**Health care cost offset** (CAD$ millions)	14,801 (13,620, 15,717)	22,747 (20,535, 24,067)	37,548 (34,155, 39,784)
**ICER**^3^ (CAD$/QALY)	-25,850 (-28,668, -23,741)	-24,369 (-26,874, -22,605)	-24,933 (-27,569, -23,043)
**Tax revenue** (CAD$ millions)	17,378 (16,889, 17,693)	26,675 (26,076, 27,028)	44,016 (43,346, 44,620)
**Intervention costs** (CAD$ millions)	348 (338, 354)	534 (522, 541)	880 (867, 892)

Values are presented as mean (95% uncertainty interval).

^1^ DALYs: Disability-adjusted life years

^2^ QALYs: Quality-adjusted life years

^3^ ICERs: Incremental cost-effectiveness ratio. All values were discounted by 1.5% to their present values (2015).

Table 1 also shows that the simulated SSB tax was projected to generate $44,016 million (95% UI: CAD$43,346, 44,620 million) tax revenue over 80 years. The costs of the government to implement the SSB tax were estimated at CAD$880 million (95% UI: CAD$867, 892 million).

### Scenario analyses

**Fig 5:**
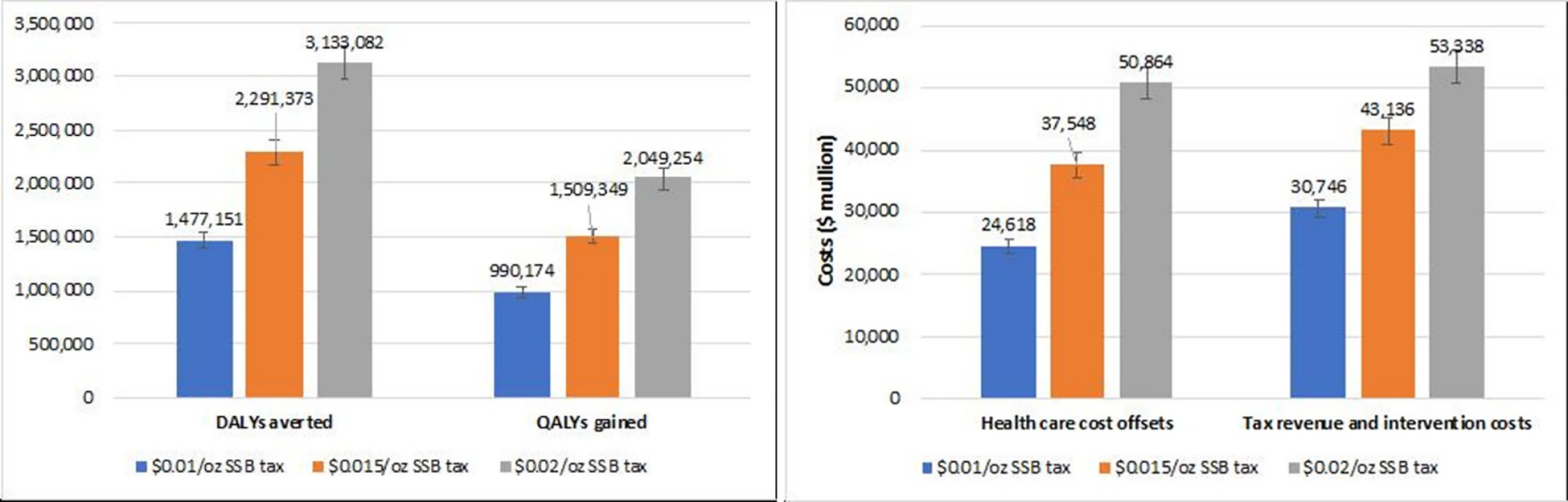
The comparison of impact of a CAD$0.01/oz, CAD$0.015/oz, and CAD$0.02/oz SSB tax for a lifetime period. (A) DALYs averted and QALYs gained; (B) Health care cost offsets, tax revenue, and intervention costs (CAD$ million). DALY: Disability-adjusted life years. QALY: quality-adjusted life years. Error bars represent 95% uncertainty intervals. Tax revenue and intervention costs: the sum of intervention costs and tax revenue.

Fig 5 shows the impact of the CAD$0.01/oz and CAD$0.02/oz SSB tax in Canada. The CAD$0.02/oz SSB tax was estimated to avert 27% more DALYs and gain 26% more QALYs than the CAD$0.015/oz SSB tax. The CAD$0.01/oz SSB tax was estimated to avert 36% fewer DALYs and gain 34% less QALY than a CAD$0.015/oz SSB tax. The combined health care cost saving, tax revenue, and intervention costs for the CAD$0.01/oz and CAD$0.02/oz SSB tax were estimated at CAD$55,364 million and CAD$104,202 million, respectively.

### Sensitivity analyses

The results of univariate sensitivity analyses with comparisons to base case tax scenarios (Table 3) show that after removing the BMI trend over time, the health benefits and health care cost offsets changed minimally. When the SSB tax did not pass to consumers completely (i.e., 80% pass-through rate), health benefits and health care cost savings would decline by around 20% accordingly. Passing more tax to consumers (i.e., 120% pass-through rate) was estimated to gain around 20% more DALYs, QALYs and health care cost offsets.

**Table 3:** Sensitivity analyses for a CAD$0.015/oz SSB tax in Canada in the lifetime period.

	Lifetime DALYs averted		Lifetime QALYs gained		Health care cost offsets (CAD$ million)		Tax revenue and intervention costs ^1^ (CAD$ million)	
**A CAD$0.015/oz SSB tax—**base case	2,291,373		1,509,349		37,548		43,136	
1) BMI remain at 2015 levels	2,291,250	-0.5%	1,514,604	-0.3%	37,333	-0.6%	42,913	-0.5%
2) Tax pass-on 80%	1,774,659	-22.6%	1,181,832	-21.7%	29,602	-21.2%	35,751	-17.1%
3) Tax pass-on 120%	2,842,662	24.1%	1,859,558	23.2%	46,085	22.7%	49,202	14.1%
4) Health gains and costs discounted by 3%	1,133,389	-50.5%	766,270	-49.2%	23,031	-38.7%	28,371	-34.2%
5) Price elasticities from the US	1,899,407	-17.1%	1,245,633	-17.5%	31,838	-15.2%	44,052	2.1%

^1^ Tax revenue and intervention costs: the sum of intervention costs and tax revenue.

The discounting rate had a substantial impact on outcomes (Table 3). There was about a 50.5% decline in DALYs averted, a 49.2% reduction in QALYs gained, and a 38.7% reduction in health care cost offset when the outcomes discount rate was increased from 1.5% to 3% in the lifetime period. Price elasticities also have unignorable impacts on outcomes of simulated sugar taxes. When we applied the US price elasticities, the outcomes of the SSB tax would decrease by around 17%.

## Discussion

This study showed that a CAD$0.015/oz SSB tax would decrease the Canadian population SSBs consumption, TEI, and BMI. These changes would reduce 282,104 cases of overweight and obesity, prevent 210,542 new cases of CDs, avert 2.3 million DALYs, and gain 1.5 million QALYs over the lifetime of the adult population. From a public health care payer’s perspective, the simulated SSB tax was cost-effective with CAD$-24,933/QALY ICER, and it would save CAD$37,548 million in health care costs. The simulated tax has the potential to generate CAD$43,136 million in net tax revenue over the lifetime period.

The findings in the current study support the conclusions of previous Canadian [19, 21] and international economic evaluations: the sugar tax on SBBs is a cost-effective policy to improve population health [18]. Compared with findings of the previous economic evaluation studies where SSB tax has the potential to avert 5,840 to 2,800,000 DALYs and save CAD$10.7 to CAD$105 billion in health care costs [18], the estimated outcomes in the present study were at a higher level. The SSB tax simulated in this study has relatively higher impacts on outcomes partly because we estimated the SSB tax for all adult populations over lifetime horizons in Canada. Some previous studies only estimated SSBs tax for the working-age population aged 25 to 64 over a shorter period (e.g., ten years) [18]. Other factors, such as tax rates, passthrough rate, and demand elasticities might also lead to the variances of the outcomes. Furthermore, consistent with the previous Canadian SSB tax evaluations [19, 21], this study indicates that males were estimated to have more absolute reductions in SSBs consumption and energy intake and gain more health benefits and cost savings than females after implementing SSB tax.

The current study is the first Canadian study to build a model that includes all CDs and own- and cross-price elasticities. The different numbers or categories of CDs included in the SSB tax model would lead to different outcomes. Compared with the previous Canadian study that used the same population and similar model structure [19, 21], the same level (20%) SBB tax simulated in this study would avert three times more DALYs and health care costs. The main reason is that we included all the RRs of diseases related to BMI and SSB consumption from the 2017 GBD studies in the simulation model [48]. Unlike many previous modelling studies of the SSB tax (including two Canadian studies [19, 21]) that only included part of related diseases [18], our estimation could avoid underestimation of the health and economic benefits of the SSB tax.

The price elasticities of SSBs are significant factors in the sugar taxes simulation. In this study, we used both the own- and cross-price elasticities of SSBs. The own-price elasticities (-1.15) showed that SSBs are elastic, and SSB tax could effectively reduce SSBs consumption. The own-price elasticities used in the current study are close to that of the previous Canadian SSB tax simulation studies (ranging from -0.87 to -1.20) [19, 21]. The cross-price elasticities reflected the substitute consumption of all untaxed foods and beverages in Canada. It helped us more accurately estimate the changes in TEI, BMI, and BMI-mediated disease risks attributed to the SSB tax. We used price elasticities from New Zealand [41] because Canada did not have comprehensive price elasticities that covered all food categories. The sensitivity analysis showed that the SSB tax remained highly cost-effective when applying price elasticities from the US (-1.04) [43]. The impact of different price elasticities on health and economic outcomes were smaller than the changes after using different pass-through and discount rates.

We assumed that the BMI of the modelled population would change following the predicted historic BMI trends [44]. The trend was estimated using the existing age and sex regression coefficient derived from the CCHS 2001–2018 [44]. Considering that BMI might not change following this trend for the next 80 years, we conducted a sensitivity analysis to test the impact of this BMI trend by removing it from the model. We also assumed that 100% of the SSB tax would be passed to the consumers and shown on the price tags. To reflect how pass-through rate changes outcomes of SSB tax, we assumed 80% and 120% tax were passed to consumers in the sensitivity analysis. This was based on the data from other countries that have implemented SSB tax and previous SSB tax simulation models [9, 33, 34]. We used a 1.5% yearly discount rate for the health and economic outcomes based on CADTH guidelines [25]. Because some economic evaluation guidelines in other countries recommend a 3% discount rate [60], we used it in the sensitivity analysis. Except for the discount rate, these assumptions had relatively small impacts and they would not change the interpretation of the results.

The SSB tax model built in this study has several strengths. We designed different levels of SSB tax in the scenario analysis to provide more information about the impact of the tax level. We estimated both DALYs and QALYs as health outcomes of sugar taxes in the model. It allows us to calculate ICERs and compare different policies and international studies. Moreover, this study conducted an age and sex-specific analysis. The results reflected that the difference in the impact of SSB tax comes from the different individual-level characteristics of consumers [20]. For example, the younger age groups showed larger changes in SSB consumption, TEI, and BMI due to the SSB tax. Additionally, we included all related CDs identified by the 2017 GBD [2] and considered both the own and cross-price elasticities of a wide range of relevant food and beverage items. Unlike in most previous Canadian and international studies, this approach avoided underestimating impacts of SSB tax.

Several limitations warrant consideration. Firstly, the price elasticities of demand from New Zealand used in the model can lead to bias. Based on the sensitivity analysis, we found that this bias is acceptable and would not impact the high cost-effectiveness of the SSB tax. We assumed the price elasticities were similar across sex and age groups, which means changes in SSB consumption in percentage terms were the same for different subgroups. The differences in SSB consumption in absolute terms were from the baseline difference in SSB consumption. We also assumed that CDs included in the model were independent of each other. Although it can avoid double counting and simplify the model structure, the assumption ignores the potentially non-additive property of disability-related comorbidities of CDs. It might lead to underestimation or overestimation of outcomes and ICERs of SSB tax. In addition, we used the 24h dietary recalls from the 2015 CCHS–Nutrition dataset, which is the best and largest survey available in Canada. However, it is prone to error as every dietary assessment method. We also assumed that the dietary pattern would change as a one-time in one year, which might result in overestimating the long-term outcomes. More future studies that estimate the long-term impact of SSB taxes on demand are needed. Additionally, we used a percentage of tax revenue as a proxy for actual administrative costs when estimating intervention costs. Empirical research evaluating the costs of implementing a SSB tax in Canada should be conducted in the future.

Furthermore, this study only used the public health care payer perspective due to lacking costs for informal health care or non-healthcare sector for each disease included in the model. More research using other perspectives, in particular societal perspective, could be conducted in the future to comprehensively reflect impact of SSB tax (e.g., indirect costs) [61]. Moreover, we did not weigh SSBs by sales level when calculating the national average SSB price because lacking sales data. It would impact the cost-effectiveness results, but it might lead to bias in estimation of tax revenue and intervention costs. Lastly, due to the data restriction of disease risks, we only estimated the health impacts of SSB taxes on Canadian adults aged 20 years and above. The current study is unable to reflect changes in DALYs, QALYs and health care costs attributed to SSB taxes for children and adolescents. Due to the very low prevalence of CDs among those age groups, the impact of this age-group exclusion on the results could be negligible.

## Conclusions

The implementation of the SSB tax in Canada would be a cost-effective policy option to reduce obesity and related CDs and improve quality of life. The combination of SSB tax revenue and health care costs was much higher than intervention costs. The SSB tax simulation model that includes all related CDs identified by the 2017 GBD and the substitute effects of all untaxed products could be used in future research to explore the impact of other sugar tax options.

## Supporting information

S1 File (DOCX)
