# Supplementary material for: Modelling the health and economic impact of sugary sweetened beverage tax in Canada
Source: PLoS One. 2022 Nov 10;17(11):e0277306. doi: 10.1371/journal.pone.0277306 (PMC9648777; doi:10.1371/journal.pone.0277306)
Supplement: S1 File — (DOCX) [file pone.0277306.s001.docx]

**Supporting Information**

**S1 Table.** The Consolidated Health Economic Evaluation Reporting Standards (CHEERS) Checklist for the sugar tax model

| **Item** | **Item No.** | **Recommendation** | **Reported on page No/** para **No** |
| --- | --- | --- | --- |
| **Title and abstract** |  |  |  |
| Title | 1 | Identify the study as an economic evaluation or use more specific terms such as “cost-effectiveness analysis”, and describe the interventions compared. | Title |
| Abstract | 2 | Provide a structured summary of objectives, perspective, setting, methods (including study design and inputs), results (including base case and uncertainty analyses), and conclusions. | Abstract |
| **Introduction** |  |  |  |
| Background and objectives | 3 | Provide an explicit statement of the broader context for the study.  Present the study question and its relevance for health policy or practice decisions. | Introduction |
| **Methods** |  |  |  |
| Target population and subgroups | 4 | Describe characteristics of the base case population and subgroups analyzed, including why they were chosen. | Method para 2 |
| Setting and location | 5 | State relevant aspects of the system(s) in which the decision(s) need(s) to be made. | Method para 1 |
| Study perspective | 6 | Describe the perspective of the study and relate this to the costs being evaluated. | Method para 1 |
| Comparators | 7 | Describe the interventions or strategies being compared and state why they were chosen. | Method para 1，4 |
| Time horizon | 8 | State the time horizon(s) over which costs and consequences are being evaluated and say why appropriate. | Method para 1 |
| Discount rate | 9 | Report the choice of discount rate(s) used for costs and outcomes and say why appropriate. | Method para 1 |
| Choice of health outcomes | 10 | Describe what outcomes were used as the measure(s) of benefit in the evaluation and their relevance for the type of analysis performed. | Method para 1 |
| Measurement of effectiveness | 11a | Single study-based estimates: Describe fully the design features of the single effectiveness study and why the single study was a sufficient source of clinical effectiveness data. | Method para 3 |
|  | 11b | Synthesis-based estimates: Describe fully the methods used for identification of included studies and synthesis of clinical effectiveness data. | N/A |
| Measurement and valuation of preference-based outcomes | 12 | If applicable, describe the population and methods used to elicit preferences for outcomes. | Method para 14, 15 |
| Estimating resources and costs | 13a | Single study-based economic evaluation: Describe approaches used to estimate resource use associated with the alternative interventions. Describe primary or secondary research methods for valuing each resource item in terms of its unit cost.  Describe any adjustments made to approximate to opportunity costs. | N/A |
|  | 13b | Model-based economic evaluation: Describe approaches and data sources used to estimate resource use associated with model health states. Describe primary or secondary research methods for valuing each resource item in terms of its unit cost. Describe any adjustments made to approximate to opportunity costs. | Method para 16, 17 |
| Currency, price date, and conversion | 14 | Report the dates of the estimated resource quantities and unit costs. Describe methods for adjusting estimated unit costs to the year of reported costs if necessary. Describe methods for converting costs into a common currency base and the exchange rate. | Method para 16, 17 |
| Choice of model | 15 | Describe and give reasons for the specific type of decision analytical model used. Providing a figure to show model structure is strongly recommended. | Method para 5, 6 |
| Assumptions | 16 | Describe all structural or other assumptions underpinning the decision-analytical model. | Method para 7-14 |
| Analytical methods | 17 | Describe all analytical methods supporting the evaluation. This could include methods for dealing with skewed, missing, or censored data; extrapolation methods; methods for pooling data; approaches to validate or make adjustments (such as half cycle corrections) to a model; and methods for handling population heterogeneity and uncertainty. | Method para 14, 17 |
| **Results** |  |  |  |
| Study parameters | 18 | Report the values, ranges, references, and, if used, probability distributions for all parameters. Report reasons or sources for distributions used to represent uncertainty where appropriate.  Providing a table to show the input values is strongly recommended. | Method para 7-17, Results para 1 |
| Incremental costs and outcomes | 19 | For each intervention, report mean values for the main categories of estimated costs and outcomes of interest, as well as mean differences between the comparator groups. If applicable, report incremental cost-effectiveness ratios. | Results para 1-4 |
| Characterizing uncertainty | 20a | Single study-based economic evaluation: Describe the effects of sampling uncertainty for the estimated incremental cost and incremental effectiveness parameters, together with the impact of methodological assumptions (such as discount rate, study perspective). | N/A |
|  | 20b | Model-based economic evaluation: Describe the effects on the results of uncertainty for all input parameters, and uncertainty related to the structure of the model and assumptions. | Results para 5-6 |
| Characterizing heterogeneity | 21 | If applicable, report differences in costs, outcomes, or cost-effectiveness that can be explained by variations between subgroups of patients with different baseline characteristics or other observed variability in effects that are not reducible by more information. | Results para 1, 2 |
| **Discussion** |  |  |  |
| Study findings, limitations, generalizability, and current knowledge | 22 | Summarize key study findings and describe how they support the conclusions reached. Discuss limitations and the generalizability of the findings and how the findings fit with current knowledge. | Discussion |
| **Other** |  |  |  |
| Source of funding | 23 | Describe how the study was funded and the role of the funder in the identification, design, conduct, and reporting of the analysis. Describe other non-monetary sources of support. | Funding |
| Conflicts of interest | 24 | Describe any potential for conflict of interest of study contributors in accordance with journal policy. In the absence of a journal policy, we recommend authors comply with International Committee of Medical Journal Editors recommendations. | Declaration of Competing Interest |

Adapted from Husereau et al. [1].**S2 Table.** Own- and cross-price food elasticities values (Means and Standard Errors (SE))

|  | **Non-alcoholic beverages** |
| --- | --- |
| **Non-alcoholic beverages** | -1.15 (0.02) |
| **Bread and breakfast cereals** | 0.03 (0.02) |
| **Chocolate, confectionary and snacks** | -0.08 (0.02) |
| **Cake and biscuits** | -0.05 (0.02) |
| **Prepared, preserved and processed meat** | 0.02 (0.02) |
| **Sauces, sugar and condiments** | -0.03 (0.01) |
| **Other grocery food** | -0.08 (0.05) |
| **Pasta, grains and flours** | 0.01 (0.01) |
| **Margined and edible oil** | 0.01 (0.01) |
| **Poultry** | 0.06 (0.01) |
| **Pork** | 0.05 (0.01) |
| **Beef, lamb, and** | 0.05 (0.02) |
| **Fish and seafood** | 0.03 (0.01) |
| **Vegetables** | 0.01 (0.02) |
| **Cheese and cream** | 0.05 (0.01) |
| **Milk, yogurt and eggs** | 0.09 (0.02) |
| **Fruits** | -0.01 (0.02) |
| **Pastry cook products** | -0.02 (0.01) |

From Ni Mhurchu et al. [2]

**S3 Table.** Chronic diseases included in the simulation model

| **Disease category** | **Chronic disease** |
| --- | --- |
| Diabetes | Oesophageal cancer |
| Cancer | Colon and rectum cancer |
|  | Liver cancer |
|  | Gallbladder and biliary tract cancer |
|  | Pancreatic cancer |
|  | Breast cancer ^1^ |
|  | Uterine cancer ^1^ |
|  | Ovarian cancer ^1^ |
|  | Kidney cancer |
|  | Thyroid cancer |
|  | Non-Hodgkin’s lymphoma |
|  | Multiple myeloma |
|  | Leukaemia |
| Cardiovascular diseases | Ischemic heart disease |
|  | Ischemic stroke |
|  | Hemorrhagic stroke |
|  | Hypertensive heart disease |
|  | Atrial fibrilliation and flutter |
| Other condition | Asthma |
|  | Gallbladder and biliary disease |
|  | Alzheimer’s disease and other dementias |
|  | Diabetes mellitus type 2 |
|  | Chronic kidney disease due to diabetes |
|  | Chronic kidney disease due to hypertension |
|  | Chronic kidney disease due to glomerulonephritis |
|  | Chronic kidney disease due to other causes |
|  | Cataract |
|  | Low back pain |
|  | Gout |
|  | Osteoarthritis of the hip |

^1^ Only for females.

**S4 Table.** Trend in body mass index

| **Age** | **Females** (kg/m^2^ per year) | **Males** (kg/m^2^ per year) |
| --- | --- | --- |
| 20~24 | 0.010 | 0.017 |
| 25~29 | 0.018 | 0.027 |
| 30~34 | 0.026 | 0.036 |
| 35~39 | 0.033 | 0.044 |
| 40~44 | 0.039 | 0.051 |
| 45~49 | 0.044 | 0.057 |
| 50~54 | 0.049 | 0.063 |
| 55~59 | 0.054 | 0.068 |
| 60~64 | 0.057 | 0.072 |
| 65~69 | 0.060 | 0.075 |
| 70~74 | 0.063 | 0.078 |
| 75~79 | 0.065 | 0.080 |
| 80+ | 0.066 | 0.082 |

From Lau et al. [3].

**S5 Table.** Disease data sources and processing notes for sugar tax model

| **Disease** | **Data Source** | **Notes** |
| --- | --- | --- |
| Oesophageal cancer | Incidence rates from the Statistics Canada (Table 13-10-0111-01 (2015)); Death cases from Statistic Canada (Table: 13-10-0142-01 (2015)); Prevalence cases from GBD Result Tool (2015) | Prevalence rates from Statistic Canada are not available for 5-age group |
| Colon and rectum cancer | Incidence rates from the Statistics Canada (Table 13-10-0111-01 (2015)); Death cases from Statistic Canada (Table: 13-10-0142-01 (2015)); Prevalence cases from GBD Result Tool (2015) | Prevalence rates from Statistic Canada are not available for 5-age group |
| Liver cancer | Incidence rates from the Statistics Canada (Table 13-10-0111-01 (2015)); Death cases from Statistic Canada (Table: 13-10-0142-01 (2015)); Prevalence cases from GBD Result Tool (2015) | Prevalence rates from Statistic Canada are not available for 5-age group |
| Gallbladder and biliary tract cancer | Incidence cases from GBD Result Tool (2015); Death cases from Statistic Canada (Table: 13-10-0142-01 (2015)); Prevalence cases from GBD Result Tool (2015) | Incidence rates from Statistic Canada are not available for biliary cancer; Prevalence rates from Statistic Canada are not available for 5-age group |
| Pancreatic cancer | Incidence rates from the Statistics Canada (Table 13-10-0111-01 (2015)); Death cases from Statistic Canada (Table: 13-10-0142-01 (2015)); Prevalence cases from GBD Result Tool (2015) | Prevalence rates from Statistic Canada are not available for 5-age group |
| Breast cancer | Incidence rates from the Statistics Canada (Table 13-10-0111-01 (2015)); Death cases from Statistic Canada (Table: 13-10-0142-01 (2015)); Prevalence cases from GBD Result Tool (2015) | Prevalence rates from Statistic Canada are not available for 5-age group |
| Uterine cancer | Incidence rates from the Statistics Canada (Table 13-10-0111-01 (2015)); Death cases from Statistic Canada (Table: 13-10-0142-01 (2015)); Prevalence cases from GBD Result Tool (2015) | Prevalence rates from Statistic Canada are not available for 5-age group |
| Ovarian cancer | Incidence rates from the Statistics Canada (Table 13-10-0111-01 (2015)); Death cases from Statistic Canada (Table: 13-10-0142-01 (2015)); Prevalence cases from GBD Result Tool (2015) | Prevalence rates from Statistic Canada are not available for 5-age group |
| Kidney cancer | Incidence rates from the Statistics Canada (Table 13-10-0111-01 (2015)); Death cases from Statistic Canada (Table: 13-10-0142-01 (2015)); Prevalence cases from GBD Result Tool (2015) | Prevalence rates from Statistic Canada are not available for 5-age group |
| Thyroid cancer | Incidence rates from the Statistics Canada (Table 13-10-0111-01 (2015)); Death cases from Statistic Canada (Table: 13-10-0142-01 (2015)); Prevalence cases from GBD Result Tool (2015) | Prevalence rates from Statistic Canada are not available for 5-age group |
| Non-Hodgkin’s lymphoma | Incidence rates from the Statistics Canada (Table 13-10-0111-01 (2015)); Death cases from Statistic Canada (Table: 13-10-0142-01 (2015)); Prevalence cases from GBD Result Tool (2015) | Prevalence rates from Statistic Canada are not available for 5-age group |
| Multiple myeloma | Incidence rates from the Statistics Canada (Table 13-10-0111-01 (2015)); Death cases from Statistic Canada (Table: 13-10-0142-01 (2015)); Prevalence cases from GBD Result Tool (2015) | Prevalence rates from Statistic Canada are not available for 5-age group |
| Leukaemia | Incidence rates from the Statistics Canada (Table 13-10-0111-01 (2015)); Death cases from Statistic Canada (Table: 13-10-0142-01 (2015)); Prevalence cases from GBD Result Tool (2015) | Prevalence rates from Statistic Canada are not available for 5-age group |
| Ischemic heart disease | Incidence cases from GBD Result Tool (2015); Death cases from Statistic Canada (Table: 13-10-0147-01 (2015)); Prevalence cases from GBD Result Tool (2015) | Incidence and prevalence rates from Statistic Canada are not available for people aged <20 and 5-age group |
| Ischemic stroke | Incidence cases from GBD Result Tool (2015); Death cases from GBD Result Tool (2015); Prevalence cases from GBD Result Tool (2015) | Incidence and prevalence rates from Statistic Canada are not available for people aged <20 years old and 5-age group; Death cases from Statistic Canada are not available for ischemic stroke |
| Hemorrhagic stroke | Incidence cases from GBD Result Tool (2015); Death cases from GBD Result Tool (2015); Prevalence cases from GBD Result Tool (2015) | Incidence and prevalence rates from Statistic Canada are not available for people aged <20 years old and 5-age group; Death cases from Statistic Canada are not available for ischemic stroke |
| Hypertensive heart disease | Death cases from GBD Result Tool (2015); Prevalence cases from GBD Result Tool (2015) | Incidence rates are not available from Statistic Canada and the GBD Result Tool |
| Atrial fibrilliation and flutter | Incidence cases from GBD Result Tool (2015); Death cases from Statistic Canada (Table: 13-10-0147-01 (2015)); Prevalence cases from GBD Result Tool (2015) | Incidence and prevalence rates from Statistic Canada are not available for people aged <20 and 5-age group |
| Asthma | Incidence cases from GBD Result Tool (2015); Death cases from Statistic Canada (Table: 13-10-0782-01 (2015)); Prevalence cases from GBD Result Tool (2015) | Incidence and prevalence cases from Statistic Canada are not available 5-age group |
| Gallbladder and biliary disease | Incidence cases from GBD Result Tool (2015); Death cases from GBD Result Tool (2015); Prevalence cases from GBD Result Tool (2015) | Incidence and prevalence cases from Statistic Canada are not available 5-age group; Death cases from Statistic Canada are not available for asthma |
| Alzheimer’s disease and other dementias | Incidence cases from GBD Result Tool (2015); Death cases from Statistic Canada (Table: 13-10-0145-01 (2015)); Prevalence cases from GBD Result Tool (2015) | Incidence and prevalence cases from Statistic Canada are not available 5-age group |
| Diabetes mellitus type 2 | Incidence cases from GBD Result Tool (2015); Death cases from Statistic Canada (Table: 13-10-0144-01 (2015)); Prevalence cases from GBD Result Tool (2015) | Incidence and prevalence cases from Statistic Canada are not available |
| Chronic kidney disease due to diabetes | Incidence cases from GBD Result Tool (2015); Death cases from GBD Result Tool (2015); Prevalence cases from GBD Result Tool (2015) | Incidence, death and prevalence cases from Statistic Canada are not available |
| Chronic kidney disease due to hypertension | Incidence cases from GBD Result Tool (2015); Death cases from Statistic Canada (Table: 13-10-0147-01 (2015)); Prevalence cases from GBD Result Tool (2015) | Incidence and prevalence cases from Statistic Canada are not available |
| Chronic kidney disease due to glomerulonephritis | Incidence cases from GBD Result Tool (2015); Death cases from GBD Result Tool (2015); Prevalence cases from GBD Result Tool (2015) | Incidence, death and prevalence cases from Statistic Canada are not available |
| Chronic kidney disease due to other causes | Incidence cases from GBD Result Tool (2015); Death cases from GBD Result Tool (2015); Prevalence cases from GBD Result Tool (2015) | Incidence, death and prevalence cases from Statistic Canada are not available |
| Cataract | Death cases were inputted as 0; Prevalence cases from GBD Result Tool (2015) | Incidence and prevalence cases from Statistic Canada are not available |
| Low back pain | Incidence cases from GBD Result Tool (2015); Death cases were inputted as 0; Prevalence cases from GBD Result Tool (2015) | Incidence and prevalence cases from Statistic Canada are not available |
| Gout | Incidence cases from GBD Result Tool (2015); Death cases were inputted as 0; Prevalence cases from GBD Result Tool (2015) | Incidence and prevalence cases from Statistic Canada are not available |
| Osteoarthritis of the hip | Incidence cases from GBD Result Tool (2015); Death cases were inputted as 0; Prevalence cases from GBD Result Tool (2015) | Incidence and prevalence cases from Statistic Canada are not available |
| Osteoarthritis of the knee | Incidence cases from GBD Result Tool (2015); Death cases were inputted as 0; Prevalence cases from GBD Result Tool (2015) | Incidence and prevalence cases from Statistic Canada are not available |

**S6 Table.** Direct health care costs inputs for sugar tax model (2015 CAD$)

| **Sex** | | **Age** | | **Esophagus cancer** | **Colorectal cancer** | | **Liver cancer** | **Gallbladder and billiary track cancer** | | **Pancreas cancer** | **Breast cancer** | **Uterus cancer** |
| --- | --- | --- | --- | --- | --- | --- | --- | --- | --- | --- | --- | --- |
|  | |  | | $/prevalent case | $/prevalent case | | $/prevalent case | $/prevalent case | | $/prevalent case | $/prevalent case | $/prevalent case |
| Females | | <55 | | 13,101 | 12,196 | | 13,495 | 312,114 | | 48,705 | 3,296 | 7,414 |
|  | | 55-64 | | 14,099 | 8,689 | | 11,541 | 241,301 | | 32,413 | 2,084 | 3,156 |
|  | | 65-74 | | 14,675 | 7,633 | | 12,284 | 189,180 | | 30,052 | 1,535 | 2,338 |
|  | | 75+ | | 13,645 | 8,311 | | 10,052 | 53,740 | | 16,678 | 1,396 | 4,391 |
| Males | <55 | | 22,689 | | 12,042 | 24,917 | | 152,411 | 51,650 | | - | - |
|  | 55-64 | | 23,614 | | 9,447 | 17,449 | | 75,624 | 34,734 | | - | - |
|  | 65-74 | | 26,570 | | 7,644 | 15,230 | | 99,707 | 34,130 | | - | - |
|  | 75+ | | 26,872 | | 9,229 | 19,062 | | 52,811 | 16,532 | | - | - |
| **Sex** | | **Age** | | **Ovary cancer** | **Kidney cancer** | | **Thyroid cancer** | **Non-Hodgkin lymphoma** | | **Multiple myeloma** | **Leukemia** | **Ischemic heart disease** |
|  | |  | | $/prevalent case | $/prevalent case | | $/prevalent case | $/prevalent case | | $/prevalent case | $/prevalent case | $/prevalent case |
| Females | | <55 | | 4,989 | 9,541 | | 15,640 | 21,278 | | 19,321 | 48,775 | 5,727 |
|  | | 55-64 | | 2,481 | 7,236 | | 5,852 | 11,332 | | 34,524 | 8,031 | 5,759 |
|  | | 65-74 | | 1,589 | 7,586 | | 7,081 | 7,136 | | 23,232 | 4,199 | 4,554 |
|  | | 75+ | | 4,547 | 12,381 | | 14,721 | 4,251 | | 16,194 | 9,795 | 4,260 |
| Males | | <55 | | - | 4,661 | | 17,894 | 21,887 | | 28,602 | 18,955 | 12,816 |
|  | | 55-64 | | - | 4,400 | | 5,137 | 9,126 | | 36,169 | 9,287 | 8,959 |
|  | | 65-74 | | - | 3,959 | | 6,782 | 4,962 | | 13,702 | 4,913 | 5,834 |
|  | | 75+ | | - | 6,579 | | 10,355 | 3,988 | | 12,305 | 4,843 | 5,098 |
| **Sex** | | **Age** | | **Ischemic stroke** | **Hemorrhagic stroke** | | **Hypertensive heart disease** | **Atrial fibrilliation and flutter** | | **Asthma** | **Gallbladder and biliary tract** | **Alzheimer's disease** |
|  | |  | | $/prevalent case | $/prevalent case | | $/prevalent case | $/prevalent case | | $/prevalent case | $/prevalent case | $/prevalent case |
| Females | | <55 | | 2,084 | 4,759 | | 88,473 | 344 | | 1,906 | 689 | 554 |
|  | | 55-64 | | 2,274 | 3,978 | | 124,217 | 244 | | 1,638 | 592 | 1,513 |
|  | | 65-74 | | 2,753 | 5,428 | | 60,866 | 294 | | 1,537 | 640 | 1,514 |
|  | | 75+ | | 5,234 | 14,373 | | 6,888 | 572 | | 1,467 | 896 | 1,395 |
| Males | | <55 | | 3,243 | 6,887 | | 134,425 | 213 | | 2,062 | 1,434 | 443 |
|  | | 55-64 | | 3,995 | 8,063 | | 204,777 | 265 | | 2,157 | 1,113 | 1,047 |
|  | | 65-74 | | 3,525 | 13,068 | | 83,029 | 232 | | 1,841 | 925 | 1,367 |
|  | | 75+ | | 5,876 | 25,127 | | 9,620 | 451 | | 2,087 | 1,125 | 2,329 |
| **Sex** | | **Age** | | **Diabetes** | **Chronic kidney disease** | | **Cataract** | **Low back pain** | | **Gout** | **Arthrosis of hip** | **Arthrosis of knee** |
|  | |  | | $/prevalent case | $/prevalent case | | $/prevalent case | $/prevalent case | | $/prevalent case | $/prevalent case | $/prevalent case |
| Females | | <55 | | 7,446 | 626 | | 11,269 | 782 | | 47 | 1,657 | 470 |
|  | | 55-64 | | 5,712 | 375 | | 19,107 | 602 | | 34 | 1,992 | 765 |
|  | | 65-74 | | 4,500 | 318 | | 20,645 | 544 | | 48 | 2,172 | 817 |
|  | | 75+ | | 3,931 | 280 | | 6,812 | 732 | | 124 | 2,314 | 718 |
| Males | | <55 | | 7,205 | 698 | | 12,638 | 1,124 | | 43 | 2,055 | 417 |
|  | | 55-64 | | 7,134 | 529 | | 19,904 | 966 | | 36 | 2,248 | 739 |
|  | | 65-74 | | 5,206 | 514 | | 22,019 | 702 | | 47 | 2,169 | 871 |
|  | | 75+ | | 4,245 | 391 | | 10,540 | 810 | | 107 | 2,110 | 849 |

**S7 Table.** Mean change in body mass index due to a CAD$0.015/oz SSB tax, by age and gender

| **Age group^2^** | **Females** | | **Males** | |
| --- | --- | --- | --- | --- |
|  | Mean (kg/m^2^) | 95%UI^1^ | Mean (kg/m^2^) | 95%UI |
| **1~9** | -0.07 | (-0.08, -0.07) | -0.08 | (-0.08, -0.08) |
| **10~19** | -0.12 | (-0.12, -0.11) | -0.14 | (-0.15, -0.13) |
| **20~29** | -0.16 | (-0.17, -0.14) | -0.22 | (-0.24, -0.20) |
| **30~39** | -0.15 | (-0.16, -0.13) | -0.20 | (-0.22, -0.19) |
| **40~49** | -0.11 | (-0.13, -0.10) | -0.16 | (-0.18, -0.15) |
| **50~59** | -0.13 | (-0.14, -0.12) | -0.14 | (-0.15, -0.13) |
| **60~69** | -0.12 | (-0.13, -0.10) | -0.14 | (-0.15, -0.12) |
| **70~79** | -0.13 | (-0.14, -0.11) | -0.12 | (-0.13, -0.10) |
| **80~89** | -0.15 | (-0.17, -0.14) | -0.15 | (-0.17, -0.13) |
| **90+** | -0.13 | (-0.17, -0.09) | -0.17 | (-0.24, -0.10) |
| **Total** | -0.14 | (-0.15, -0.14) | -0.18 | (-0.19, -0.18) |

^1^ 95%UI, 95% uncertainty interval; ^2^ 1~19 age groups were not included in cost-effectiveness modeling.

**S8 Table.** Prevented disease incident cases, prevalent cases, and deaths due to a CAD$0.015/oz SSB tax in Canada

|  | **Incident cases (2016-2041)** | | **Prevalent cases (2041)** | | **Deaths (2016-2041)** | |
| --- | --- | --- | --- | --- | --- | --- |
|  | Mean | 95%UI^1^ | Mean | 95%UI | Mean | 95%UI |
| **Diabetes mellitus type 2** | 72,673 | (69,866, 74,923) | 69,180 | (66,505, 71,327) | 81 | (78, 84) |
| **Cancer** | | | | | | |
| Breast cancer (premenopausal)^2^ | 1,451 | (1,186, 1,708) | 555 | (336, 765) | 132 | (102, 161) |
| Thyroid cancer | 522 | (477, 567) | 499 | (455, 541) | 1 | (1, 1) |
| Uterine cancer^2^ | 253 | (246, 260) | 222 | (216, 229) | 20 | (20, 21) |
| Colon and rectum cancer | 233 | (223, 243) | 174 | (166, 181) | 50 | (47, 52) |
| Kidney cancer | 204 | (193, 215) | 186 | (175, 196) | 8 | (7, 8) |
| Non-Hodgkin's lymphoma | 87 | (73, 101) | 72 | (60, 84) | 11 | (10, 13) |
| Esophageal cancer | 32 | (26, 39) | 11 | (9, 13) | 21 | (17, 25) |
| Liver cancers | 32 | (28, 36) | 6 | (5, 7) | 26 | (22, 29) |
| Ovarian cancer^2^ | 16 | (12, 19) | 12 | (10, 15) | 2 | (2, 3) |
| Pancreatic cancer | 13 | (11, 15) | 3 | (3, 4) | 9 | (8, 11) |
| Multiple myeloma, leukemia | 11 | (9, 13) | 9 | (8, 11) | 1 | (1, 2) |
| Gallbladder and biliary tract cancer | 7 | (7, 8) | 5 | (4, 5) | 2 | (2, 2) |
| **Cardiovascular disease** | | | | | | |
| Ischaemic heart disease | 27,972 | (23,767, 31,289) | 25,292 | (21,389, 28,307) | 1,535 | (1,274, 1,730) |
| Hemorrhagic stroke | 1,785 | (1,716, 1,845) | 1,538 | (1,489, 1,589) | 189 | (181, 196) |
| Ischaemic stroke | 1,526 | (1,448, 1,603) | 1,455 | (1,379, 1,529) | 9 | (9, 10) |
| Atrial fibrillation and flutter | 1,020 | (936, 1,099) | 956 | (878, 1,031) | 0 | (0, 0) |
| Hypertensive heart disease | 113 | (107, 119) | 38 | (36, 40) | 74 | (70, 78) |
| **Other conditions** | | | | | | |
| Gallbladder and biliary diseases | 42,055 | (39,687, 44,513) | 40,887 | (38,587, 43,280) | 5 | (5, 6) |
| low back pain | 19,679 | (18,739, 20,606) | 18,610 | (17,697, 19,518) | - | - |
| Gout | 19,396 | (17,510, 21,420) | 18,693 | (16,797, 20,549) | - | - |
| Osteoarthritis of knee | 9,842 | (9,061, 10,613) | 9,292 | (8,551, 10,024) | - | - |
| Asthma | 9,289 | (8,654, 9,982) | 8,425 | (7,790, 9,093) | 4 | (4, 4) |
| Chronic kidney disease due to other causes | 1,428 | (1,222, 1,621) | 1,318 | (1,127, 1,498) | 3 | (2, 3) |
| Osteoarthritis of hip | 387 | (352, 420) | 362 | (330, 394) | - | - |
| Chronic kidney disease due to glomerulonephritis | 155 | (130, 178) | 140 | (117, 162) | 2 | (1, 2) |
| Cataract | 119 | (105, 135) | 116 | (102, 131) | - | - |
| Chronic kidney disease due to hypertension | 106 | (91, 120) | 98 | (84, 111) | 0 | (0, 0) |
| Chronic kidney disease due to diabetes mellitus | 72 | (62, 82) | 62 | (54, 71) | 4 | (3, 5) |
| Alzheimer's disease and other dementias | 64 | (51, 77) | 200 | 159, 239) | 0 | (0, 1) |

^1^ 95%UI: 95% uncertainty interval, ^2^ Only for females.

**References**

1. Husereau DH, Drummond M, MPhil SP, Carswell C, Monher D, et al. Consolidated Health Economic Evaluation Reporting Standards (CHEERS) statement. Int J Technol Assess Health Care. 2013. 11 (6): e1-6. [https://doi.org/10.1016/j.jval.2013.02.002](about:blank)
2. Ni Mhurchu C, Eyles H, Schilling C, Yang Q, Kaye-Blake W, GencË M, et al. Food prices and consumer demand: differences across income levels and ethnic groups. PLoS ONE. 2013; 8(10):e75934.
3. Lau PW, Barendregt JJ, Veerman JL. Projecting the burden of the increasing body mass index trend in Canada over the next 25 Years. Can J Diabetes. 2013; 37:S244.
